# A Case of Malformation in the Genital Organs of a Child

**Published:** 1854-07

**Authors:** William J. Chenoweth

**Affiliations:** Darwin, Ill.


### ART. III.—A CASE OF MALFORMATION IN THE GENITAL ORGANS OF A CHILD.

BY WILLIAM J. CHENOWETH, M. D., OF DARWIN, ILL.

Mrs. B., a very delicate woman, for more than a month before she was confined with her seventh child, was troubled with cramps and false pains, for which aperients and opiates were given with temporary relief. On the 20th of April she was delivered, after a labor of about eight hours, of a large healthy-looking, very plump child. Before I saw the child I remarked to the mother that she had a fine boy, but the touch did not entirely satisfy me, and on looking at the child as the nurse received it, I expressed surprise to find that it was a girl. In a few minutes one of the ladies present called my attention to an unnatural position of the cord. We now examined the child more closely and found the external genitals presenting such an unusual appearance that the gender could not be determined. The external labia were very large, being more than two and a half inches in length, extending high up into the groin on either side, and quite prominent. They were disconnected throughout their whole extent. About an inch above their lower extremity and beneath them were two small ear-like appendages answering to the nymphæ; these were not over half an inch in length and about one-fourth of an inch in width. On separating these was seen a small tubercle, somewhat resembling the glans penis, with a small orifice; this I took to be a rudimentary penis and it contained the only aperture I could at the time discover. Below this was a small depression answering to the vagina, probably eight lines in depth. Above the nymphæ and between the external labia was a large vascular tumor, one inch and a half across the abdomen and one inch vertically, and standing out from its attachment about the same distance. This mass separated the funis from the rudimentary penis, and seemed to be placed directly in front of it, for on pulling the tumor forward the origin of the cord could not be seen. The umbilicus could not therefore have been more than from one-half to three-fourths of an inch from the penis.

The next day after the birth of the child the tumor commenced bleeding; when examined it presented a spongy appearance and blood seemed to exude from its entire surface. Solid nitrate of silver arrested the hemorrhage very promptly, but in about two hours it had to be re-applied over the whole surface; for about thirty hours the bleeding at times was quite profuse, and there was a constant oozing of blood from that portion next to the body that could not be reached by the caustic. I supposed the child would die and some of the friends thought that no effort ought to be made to save it, but I continued to apply various remedies and finally resorted to PAGLIANI's styptic, with the immediate and total arrest of the hemorrhage.

I did not see the child afterwards until the 9th of June. It is now a fine looking, sprightly, healthy child; the tumor presents a globular appearance and is about the size of a partridge egg, and has, what I did not before discover, two outlets at its lower extremity, one on the left, from an even surface; the other on the right has a small projection much like a prepuce, and urine escapes from both of these foramina at the same time, and I believe also from the small tubercle at first thought to be the penis. I could not discover where the cord had come from the abdomen, there being no mark or depression to indicate it. The anus makes its appearance at least an inch in front of its proper position, being near the place where the vulva of the female infant is naturally found.

From the prominence of the external labia, I think that each contains a testis as in Prof. GROSS' case, reported in the number of the *American Journal of Medical Science*, Oct. 1852. The tumor is probably a malformed penis and the part I first supposed to be the penis a small clitoris, as below it is a depression answering to the vagina.

The same question here arises as in Prof. GROSS' case, and with my present feelings, if the child had passed its second summer, I would urge a removal of the testes as the best possible means of "ameliorating the condition of this unfortunate" being.

